# Supplementary material for: Extracellular vesicle-derived circCEBPZOS attenuates postmyocardial infarction remodeling by promoting angiogenesis via the miR-1178-3p/PDPK1 axis
Source: Commun Biol. 2023 Feb 1;6:133. doi: 10.1038/s42003-023-04505-x (PMC9892031; doi:10.1038/s42003-023-04505-x)
Supplement: Supplementary file 2 — Supplemental Material [file 42003_2023_4505_MOESM2_ESM.pdf]

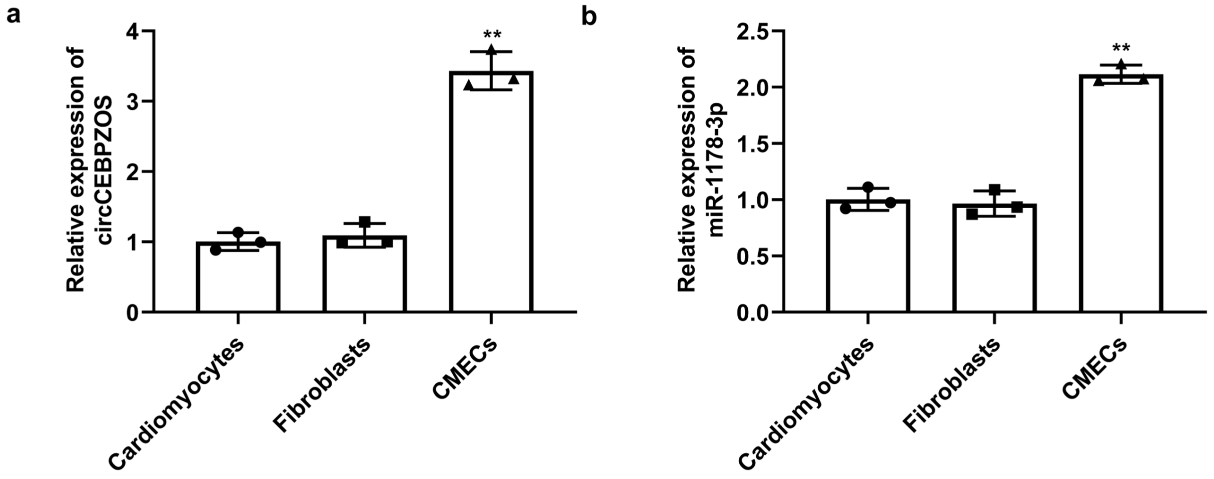

Supplementary Figure 1

The expression of circCEBPZOS and miR-1178-3p was detected by qRT-PCR in CMECs, cardiomyocytes and fibroblasts.

A and B, The expression of circCEBPZOS and miR-1178-3p was detected by qRT-PCR in CMECs, cardiomyocytes and fibroblasts, respectively. The GAPDH served as control; Data are mean±SD. **, P<0.01, CMECs group vs. cardiomyocytes or fibroblasts group.

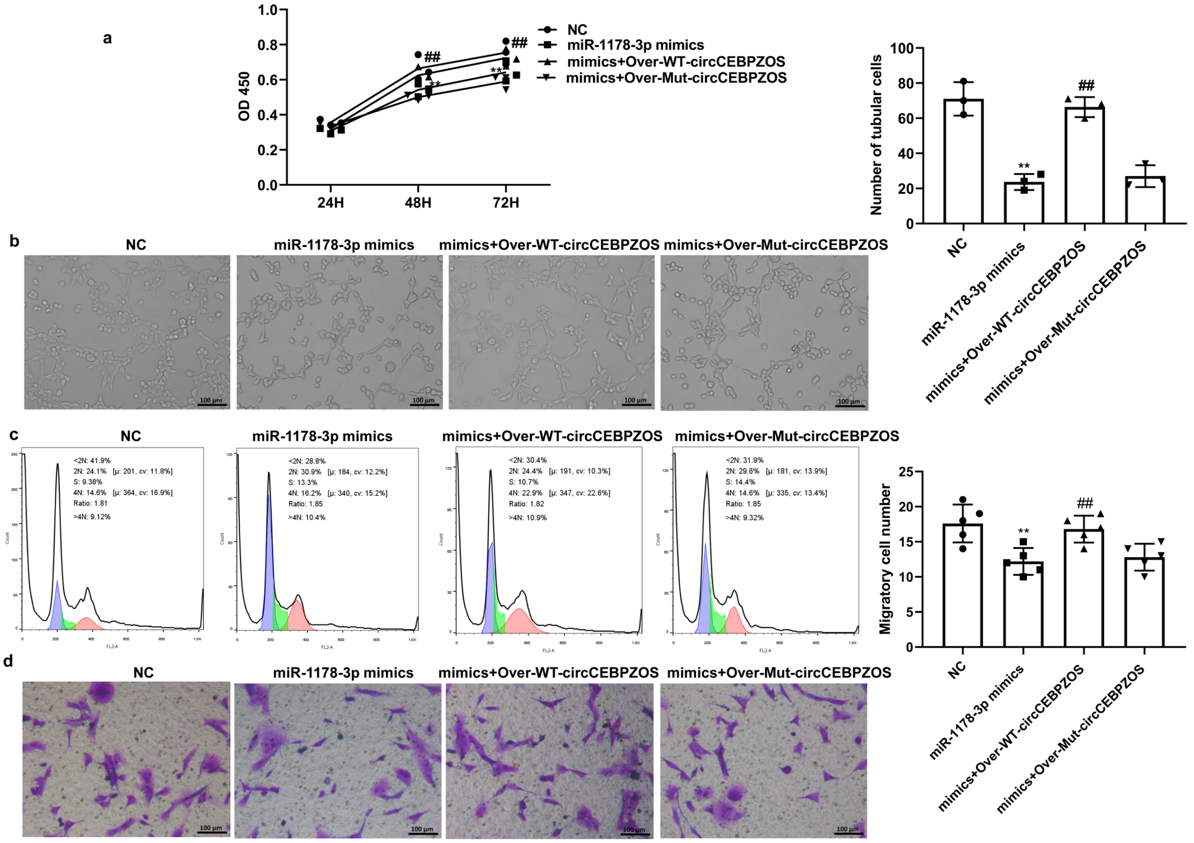

Supplementary Figure 2

The function of miR-1178-3p mimics on CMECs angiogenesis and VSMCs proliferation and migration could not be reversed by overexpression of circCEBPZOS with mutant miR-1178-3p binding site.

A, CCK-8 assay was used to detect the cell viability of VSMCs in the miR-1178-3p mimics group and rescued by circCEBPZOS overexpression with mutant miR-1178-3p binding site at 24 h, 48 h and 72 h, respectively;

B, Tube formation analysis was used to detect the capillary-like structures in the miR-1178-3p mimics group and rescued by circCEBPZOS overexpression with mutant miR-1178-3p binding site, Bar=100μm;

C, Flow cytometry was used to detect the cell cycle of VSMCs in the miR-1178-3p mimics group and rescued by circCEBPZOS overexpression with mutant miR-1178-3p binding site;

D, Migration of VSMCs was detected by Transwell assay in the circCEBPZOS overexpression group and rescued by PDPK1, Bar=200μm. Data are mean±SD. **P<0.01, Over-PDPK1 group vs. NC group; ##P<0.01, Sh-PDPK1 group vs. NC group.

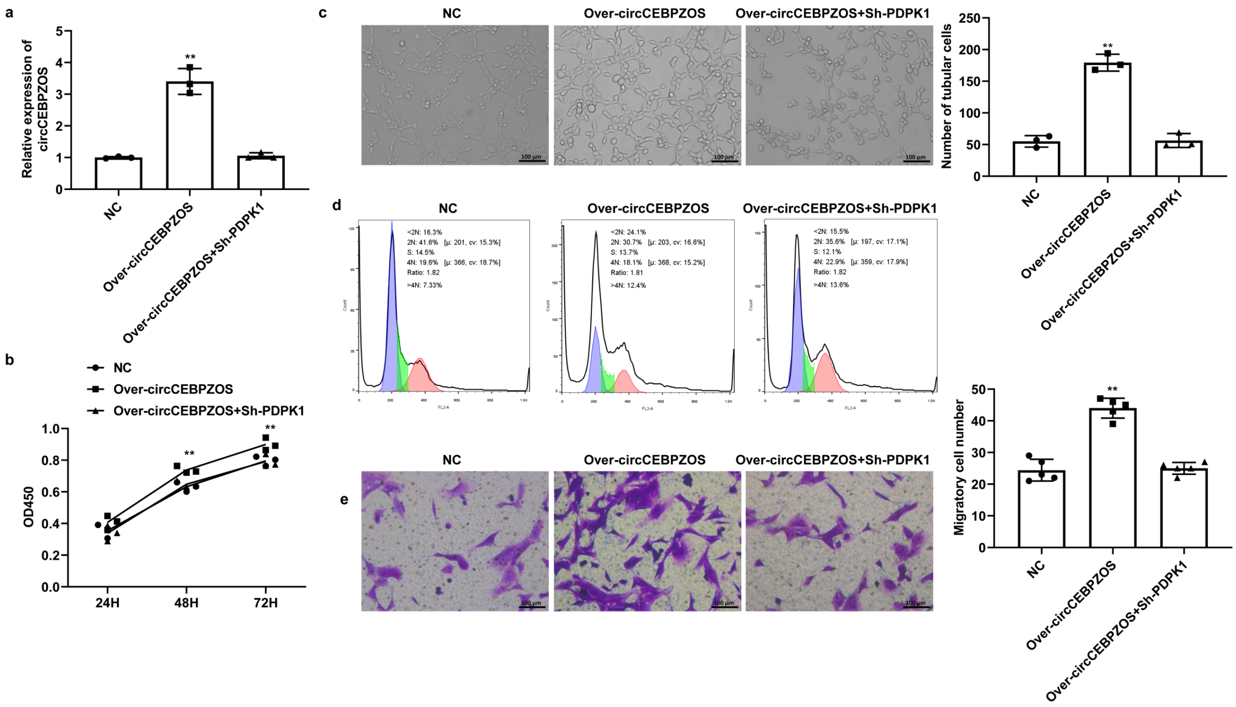

Supplementary Figure 3

The function of circCEBPZOS on CMECs angiogenesis and VSMCs proliferation and migration could be reversed by suppressing PDPK1

A, Expression of circCEBPZOS was detected by qRT-PCR, GAPDH served as control;

B, CCK-8 assay was used to detect the cell viability of VSMCs in the circCEBPZOS overexpression group and rescued by PDPK1 at 24 h, 48 h and 72 h, respectively;

C, Tube formation analysis was used to detect the capillary-like structures in circCEBPZOS overexpression group and rescued by PDPK1, Bar=100μm;

D, Flow cytometry was used to detect the cell cycle of VSMCs in the circCEBPZOS overexpression group and rescued by PDPK1;

E, Migration of VSMCs was detected by Transwell assay in the circCEBPZOS overexpression group and rescued by PDPK1, Bar=200μm. Data are mean±SD. **P<0.01, Over-PDPK1 group vs. NC group; ##P<0.01, Sh-PDPK1 group vs. NC group.

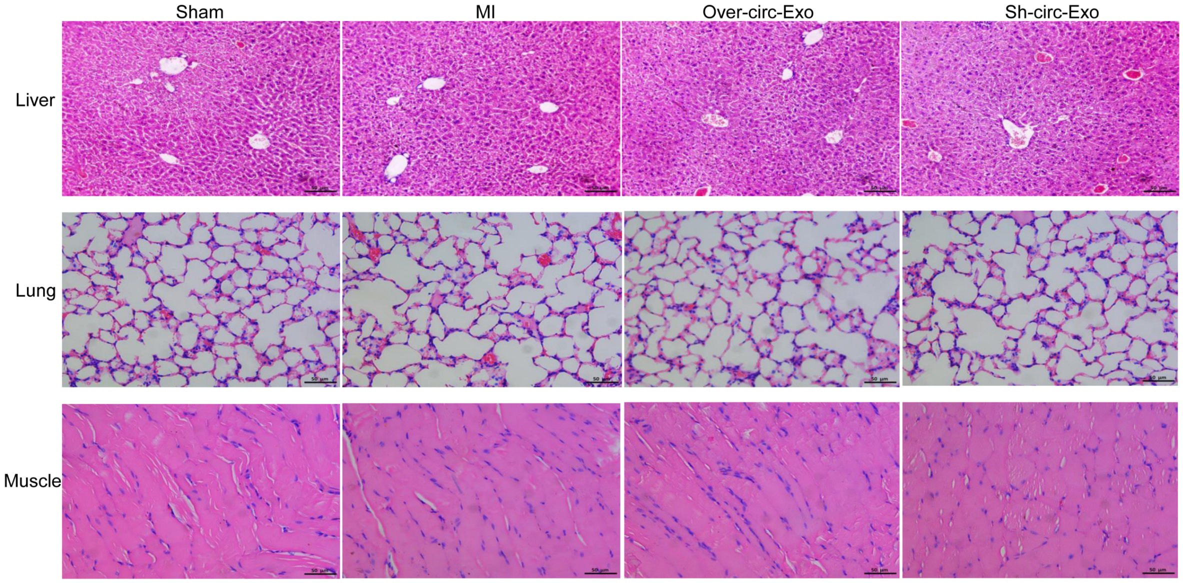

Supplementary Figure 4

H&E staining assay was used to detect the effect of exosomes to liver, lung and muscle. Bar=50μm.

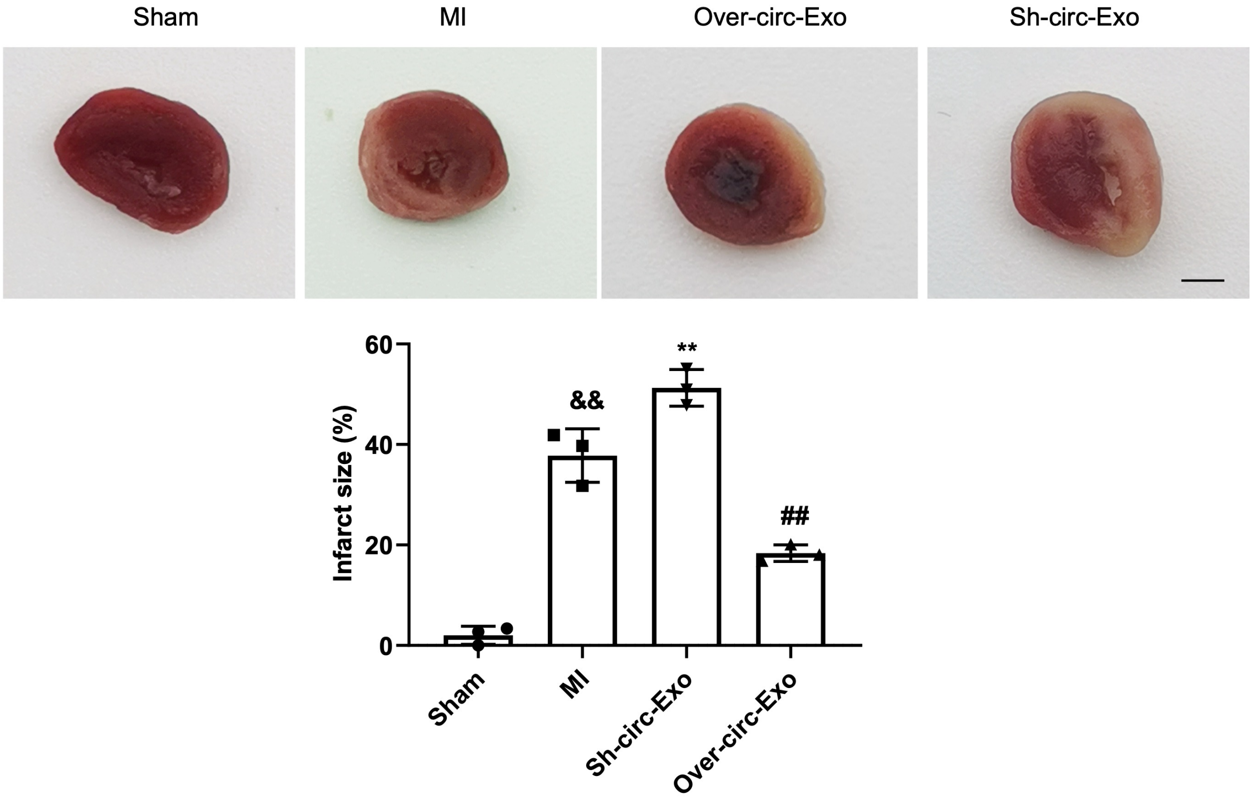

Supplementary Figure 5

Infarct size (%) was detected by 2,3,5-Triphenyltetrazolium chloride (TTC) staining in heart tissue treated with circCEBPZOS. scale bar=2mm. Data are mean±SD. **, p<0.01, Over-circCEBPZOS group vs. MI group; ##, p<0.01, sh-circCEBPZOS group vs. MI group.

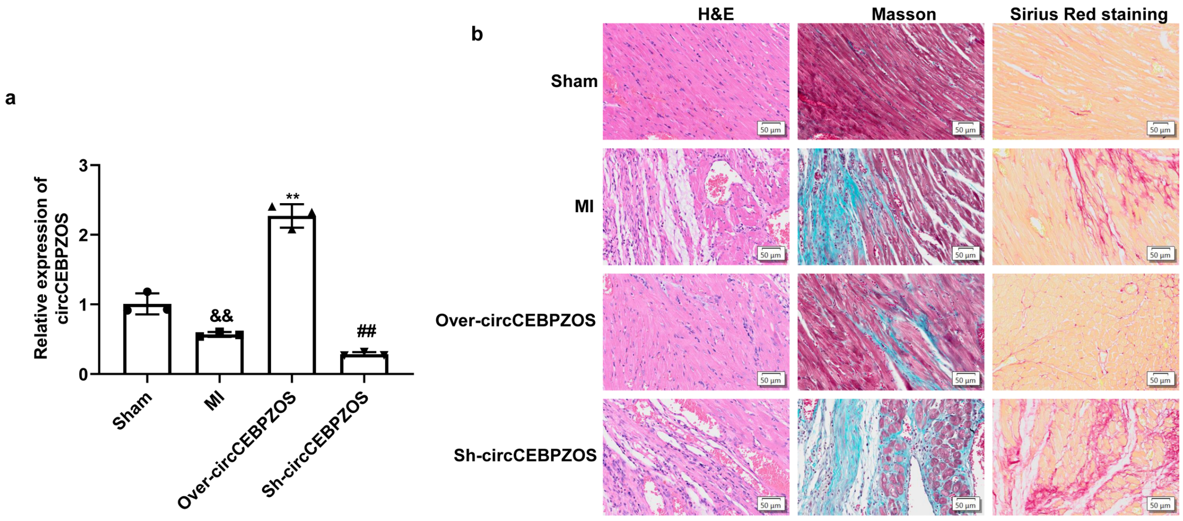

Supplementary Figure 6

circCEBPZOS alleviated postinfarct cardiac remodeling

A, qRT-PCR was used to verify the expression of circCEBPZOS in heart tissue treated with circCEBPZOS plasmid without EVs, GAPDH served as control;

B, Pathological changes detected by HE, Masson and Sirius Red staining in heart tissue treated with circCEBPZOS, Bar=50μm. Data are mean±SD. **p<0.01, Over-circCEBPZOS group vs. MI group; ##, p<0.01, sh-circCEBPZOS group vs. MI group.

Supplementary Figure 7

Uncropped and unedited blots

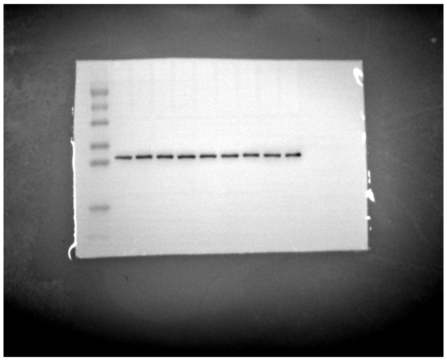

Figure1e. CD63

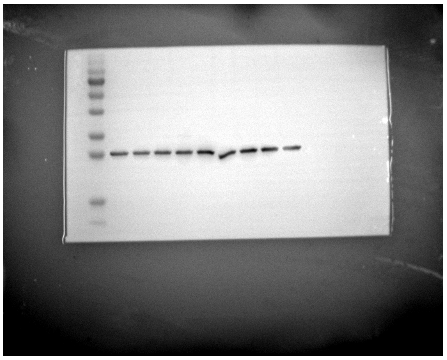

Figure1e. CD9

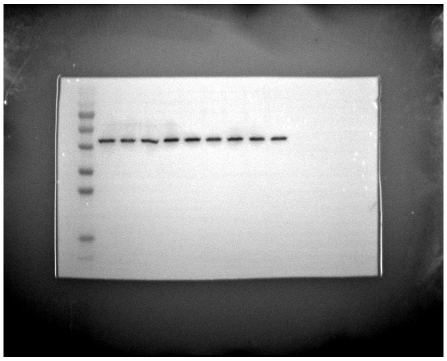

Figure1e. TSG101

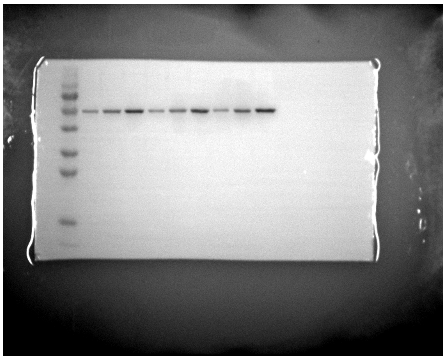

Figure 6c. CHRNB4

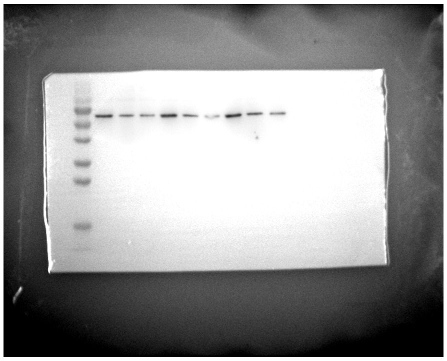

Figure 6c. PDPK1

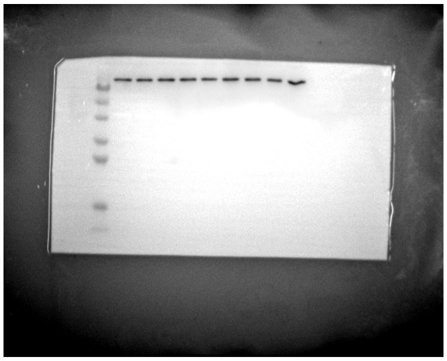

Figure 6c. STON2

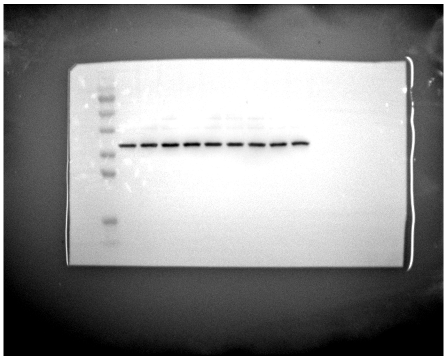

Figure 6c. GAPDH

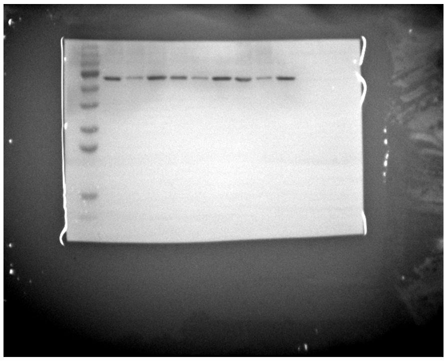
Figure 6f. PDPK1

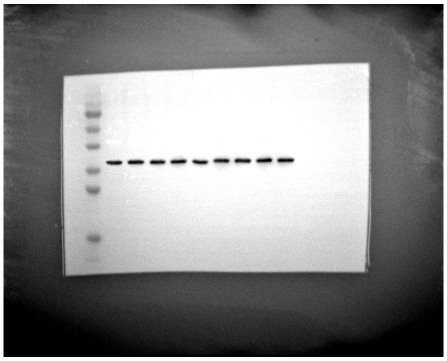
Figure 6f. GAPDH

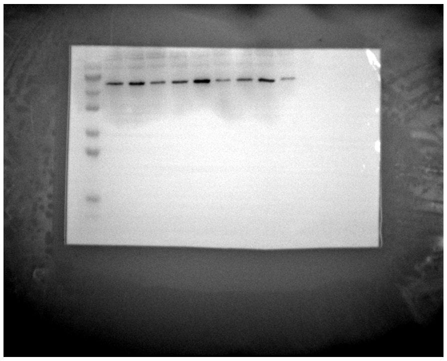

Figure 7b. PDPK1

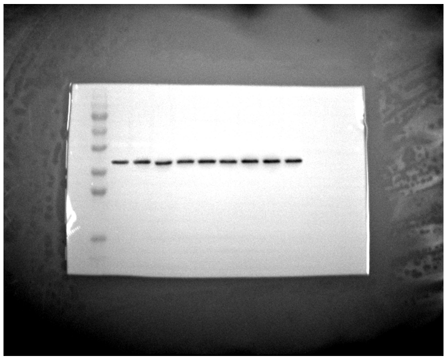

Figure 7b. GAPDH

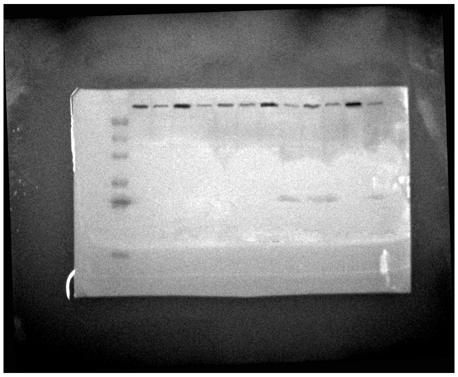

Figure 8d. HIF1α

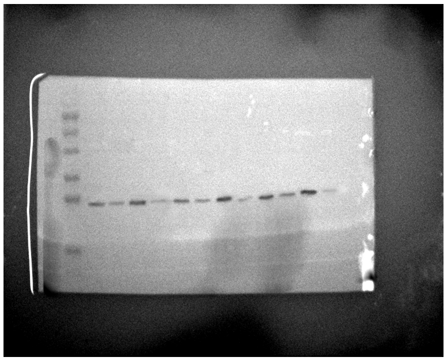

Figure 8d. VEGF

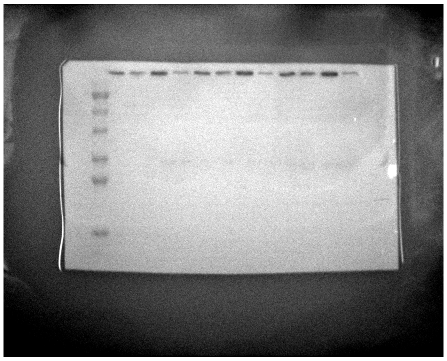

Figure 8d. VEGFR

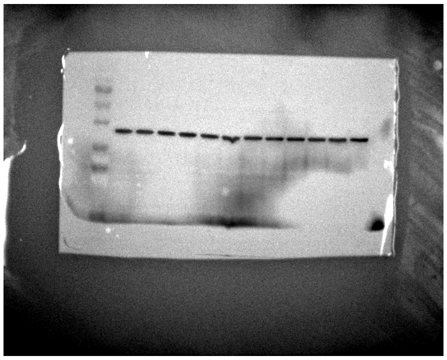

Figure 8d. GAPDH

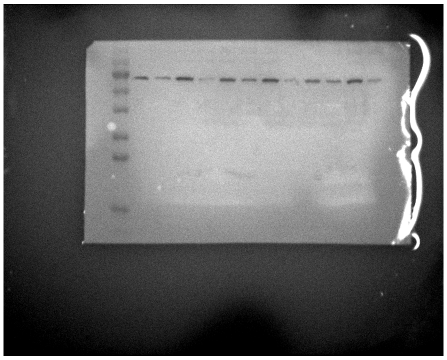

Figure 9c. PDPK1

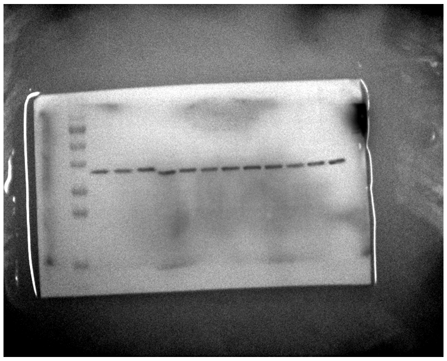

Figure 9c. GAPDH

Supplementary Tables

Supplementary Table 1. Inclusion and exclusion criteria

Echocardiographic characteristics.

	Sham	MI	Over-circ-Exo	Sh-circ-Exo
**LVEDD(mm)**	3.21±0.39	4.03±0.39*	3.79±0.42**	4.31±0.41##
**LVESD (mm)**	2.19±0.16	3.08±0.28*	2.74±0.22**	3.52±0.31##
**LVEF %**	60.51±5.65	47.49±4.85*	54.66±6.43**	37.75±4.34##
**LVFS %**	48.02±3.24	37.21±4.33*	43.19±3.94**	29.35±2.37##
**SV(μL)**	41.64±3.82	36.33±3.54*	40.65±4.32**	29.87±2.45##

LVEDD, left ventricular end-diastolic diameter; LVESD, left ventricular end-systolic diameter; FS, fractional shortening; and EF, ejection fraction. All values are mean±SE.

*p<0.01, MI-NC group vs Sham group **p<0.01, Over-circ-Exo group vs MI-NC group; ##, p<0.01, She-circ-Exo group vs MI-NC group.

Supplementary Table 2. Participant characteristics

Inclusion and exclusion criteria

**A. Inclusion criteria**	**B. Exclusion criteria**
1.Aged 18 years or over and under 80 years<br>2.First-time myocardial infarction on admission<br>3.Presence of STEMI successfully treated with coronary recanalization using PCI within 12 h after the symptom onset<br>4.Willingness to provide informed consent prior to enrollment<br>5.Able to comply with all follow-up evaluations (in the investigators opinion).<br>6.LV-remodelling defined as > 20% change in LV end-diastolic volume index [LVEDVi] assessed using echocardiography	1.Previous MI<br>2.Documented malignant arrhythmia<br>3.Severe heart failure (NYHA cardiac function class IV or left ventricular ejection fraction≤30 %) or cardiogenic shock<br>4.Serious impairment of renal function (glomerular filtration rate ≤50 mL/min per 1.73 m^2)<br>5.Hypohepatia (elevated of alanine aminotransferase and aspartate aminotransferase serum levels)<br>6.Severe coagulopathy prior to randomization<br>7.Significant neuropsychiatric condition precluding the ability to provide written informed consent<br>8.Pregnant and lactating women<br>9.Life expectancy of < 1 year

LVEDVi, left ventricular end-diastolic volume index; NYHA, New York Heart Association; PCI, percutaneous coronary intervention; STEMI, ST-elevation myocardial infarction

Supplementary Table 3. Primers used in the present study

Participant characteristics

	CR (n=10)	N-CR (n=10)	control (n=10)
Age (years)	65.3±11.2	64.2±9.3	64.5±6.8
Heart rate (beats/min)	82.1±10.5	83.3±11.2	81.4±9.4
Systolic blood pressure (mmHg)	136.4±22.4	134.8±24.5	130.1±20.1
Peak NT-proBNP (ng/L)	4452.4±321.3	201.1±64.4	23.4±11.6
LVEDVi, mL/m^2	67.3±6.5	48.9±8.3	47.6±6.9
LEVSVi, mL/m^2	34.2±8.6	31.4±6.4	30.6±7.0
LVEF, %	34.9±11.8	63.4±10.2	65.6±5.9

CR, cardiac remodelling after myocardial infarction; LVEDVi, left ventricular end-diastolic volume index; LVESVi, left ventricular end-systolic volume index; LVEF, left ventricular ejection fraction; N-CR, non-cardiac remodelling after myocardial infarction; NT-proBNP, N-terminal pro-brain natriuretic peptide.

Supplementary Table 4. Echocardiographic characteristics

Gene	Primers	Sequence (5’-3')	Tm	Product (bp)
hsa_circ_0000212	Divergent-F	CTGGTCGATCTTTGCTGTGG	58.92	111
	Divergent-R	AAAATTACCAAGAAGCCTCCCA	57.87	
hsa_circ_0007047	Divergent-F	TCTTGGAAGTTTATTACAAA	57.99	115
	Divergent-R	CAAAGTACGGGCCATCATCTAA	58.2	
	Convergent-F	GATGGCCCGTACTTTGGAACCA	58.19	113
	Convergent-R	GGCTTGTGTGCATCTTGCTAA	58.44	
hsa_circ_0089282	Divergent-F	TGTTGGCCTCCTCTATCGTC	58.89	105
	Divergent-R	TCGGCAGGAAATTTTCGTACT	57.95	
miR-1178-3p	RT	GTCGTATCCAGTGCAGGGTCCGAGGTATTCGCACTGGATACGACCTAGGG		
	Forward	GCGCGTTGCTCACTGTTCTT	61.3	
	Reverse	AGTGCAGGGTCCGAGGTATT	58.5	
U6	Forward	CTCGCTTCGGCAGCACA	60.42	

	Reverse	AACGCTTCACGAATTTGCGT	59.6<br>9	
miR-1178-3p mimics	Sence	UUGCUCACUGUUCUUCCCUAG		
	Antisence	CTAGGGAAGAACAGTGAGCAA		
miR-1178-3p inhibitor	Antisence	CTAGGGAAGAACAGTGAGCAA		
miR-1178-3p NC	Sence	UUUGUACUACACAAAAGUACUG		
	Antisence	CAGUACUUUUGUGUAGUACAAA		
GAPDH	Convergent-F	AAAGCCTGCCGGTGACTAAC	60.6<br>1	118
	Convergent-R	AGGAAAAGCATCACCCGGAG	60.0<br>4	
	Divergent-F	TCAAGTGGGGCGATGCTGGC		
	Divergent-R	TGCACCACCAACTGCTTAGC		
β-Actin	Forward	CCTCGCCTTTGCCGATCC	60.9	70
	Reverse	CGCGGCGATATCATCATCC	58.7<br>1	
PDPK1	Forward	TTCCGAGCTGGAAACGAGTAT	60.9	91
	Reverse	GGTCTCTTGCCTTAGGGAAGAA	61.1	
CHRNB4	Forward	AACCCGTTACAATAACCTGATCC	60	97
	Reverse	ATTCACGCTGATAAGCTGGGC	62.8	
STON2	Forward	ACCATGTGATTGCCACCCAC	62.8	132
	Reverse	AGCTCTCGGACTGGTCTGG	62.7	
PDPK1-pmiGLO	Forward	GCTAGCagatggctggtcatgcc	57.9	400
	Reverse	TCTAGAttatagtaaatggaca	58.4	
hsa_circ_0007047-<br>pmiGLO	Forward	GCTAGCGATGGCCCGTACTTTGG	59.5<br>6	246

	Reverse	TCTAGAATCTAATTTTTGCTGTT	59.3<br>4	
